# Divergent effects of sleep efficiency and sleep medication on episodic memory in mid to late life

**DOI:** 10.3389/frsle.2025.1691035

**Published:** 2026-01-12

**Authors:** Suhani Amin, Dokyung Yoon, Rahul Naveen, Yaseen El-Magharbel, Anya Vincent, Jessie Chih-Yuan Chien, Teal S. Eich

**Affiliations:** The Davis School of Gerontology, University of Southern California, Los Angeles, CA, United States

**Keywords:** aging, efficiency, episodic memory, medication, sleep

## Abstract

**Objectives:**

Different aspects of sleep quality are known to decline with age, and these changes have been shown to impact performance across multiple cognitive domains. However, despite a growing body of literature, the impact of changes to quality of sleep on episodic memory remains elusive, with some studies finding effects and others failing to find a relation.

**Methods:**

In this study, participants [*N* = 173, mean age = 65.30, range = [45–88]], completed the Pittsburgh Sleep Quality Index as well as three episodic memory tests (verbal and visual episodic memory and pattern separation).

**Results:**

We found that worse sleep efficiency was associated with worse overall episodic memory. Further, medication use had a positive effect on verbal, pattern separation, and overall episodic memory. Findings occurred in an age-dependent manner.

**Conclusions:**

These results underscore the complexity of sleep–memory interactions and suggest that certain aspects of episodic memory may be more sensitive to specific components of sleep quality than others, particularly as individuals age.

## Introduction

A large body of research has shown that episodic memory, the ability to recall specific events or experiences from one's past, including the context of when, where, and what happened, declines with age (Craik, 1994). In parallel, sleep, a critical determinant of health, wellbeing and cognitive function, also undergoes marked changes with age (Ravyts and Dzierzewski, 2024). Variations to sleep quality and architecture occur in both healthy as well as pathological aging, including in the early stages of Alzheimer's disease (AD) (Mander et al., 2017), a disease whose hallmark cognitive symptom is episodic memory loss. For instance, older adults tend to experience longer sleep latency, reduced rapid eye movement (REM) sleep and a decline in the amplitude of delta waves that comprise slow wave sleep (SWS) (Ohayon et al., 2004).

Although numerous studies have examined the relation between sleep and cognition in older adults, findings remain inconsistent, particularly with respect to how patterns of sleep impact episodic memory. For example, in a large (*N* = 20,065) pooled-cohort longitudinal study, both self-reported short ( ≤ 4 h) and long (≥10 h) sleep durations were associated with faster decline in verbal memory recall (Ma et al., 2020). Another pooled-cohort longitudinal study found negative impacts on episodic memory (measured by free recall of words, objects, and pictures) across a host of self-reported issues in sleep: very long (≥9 h) and short ( ≤ 6 h) sleep durations, excessive daytime napping (≥2 h), increased sleep latency, more frequent waking after sleep onset, and earlier wakening (Overton et al., 2024). Saint Martin et al. (2012), likewise, reported that subjective sleep quality was negatively associated with delayed recall on a verbal memory task, the Buschke Selective Reminding Task (BSRT) (Buschke, 1973). Together, these results suggest that poor sleep quality is associated with impairments in episodic memory.

On the other hand, several studies have reported that sleep impacts episodic memory in younger, but not older adults. The global measure of sleep quality from the Pittsburgh Sleep Quality Index (PSQI) (Buysse et al., 1989) was not associated with a visual episodic memory task in older adults in one recent study (Cohen et al., 2024) for example, and likewise no significant relationship between global PSQI scores and the BSRT (or measures of executive function) were found in another recent study of middle to late life adults (Siddarth et al., 2021). Bonnet's (1989) seminal study demonstrated that experimentally induced sleep fragmentation produced marked working memory and attention impairments as measured by the Wilkinson Addition and Auditory Vigilance Tests in younger adults, but not in older adults. A further study found that details of a verbal story learned before sleep initiation were recalled to the same extent by younger and older adults (Aly and Moscovitch, 2010). These results suggest that there may be a mechanism of aging that interacts with the relationship between sleep and episodic memory function. The current study sought to investigate how different aspects of sleep quality relate to episodic memory using three well validated, but distinct tests of episodic memory. By integrating subjective sleep measures within a cross-sectional design using multiple tasks of episodic memory, this study aimed to provide a more nuanced understanding of how sleep quality relates to episodic memory in community-dwelling adults from mid to late life.

## Materials and methods

### Participants

This paper presents results from 196 middle-aged and older adults, tested as of January 27th, 2025, as part of the ongoing University of Southern California (USC) SAGE study. Participants were recruited to the study from the USC Healthy Minds participant pool and the Banner Alzheimer's Institute Genematch program. All participants had a minimum of 8 years of formal education, were English speakers, had no MRI contraindications, were not using medications that impact the GABAergic system (e.g., benzodiazepines, barbiturates, vigabatrin, flumazenil, valproic acid, zolpidem, gabapentin, propofol or baclofen), and had not been formally diagnosed with a neurocognitive disorder including Mild Cognitive Impairment (MCI) or AD. Written informed consent was obtained from all participants, who were paid for their participation. The study was approved by the USC IRB (UP-21-00715).

### Measures

#### Sleep quality

Sleep quality was measured using the self-report PSQI, a widely-utilized instrument that assesses subjective sleep experience over the past month and has demonstrated acceptable internal homogeneity, test-retest reliability, and validity (Buysse et al., 1989). The PSQI consists of nineteen items and that generate seven component scores: subjective sleep quality (self-rated overall sleep quality), sleep latency (time required to fall asleep at night), sleep duration (actual sleep time), sleep efficiency (the percentage of time spent asleep relative to the time spent in bed), sleep disturbances (experience of difficulty sleeping such as sleep fragmentation or sleep disruption), use of sleeping medications (prescribed or over-the-counter medications to help with sleep), and daytime dysfunction (difficulties staying awake and maintaining enthusiasm during daily activities) (Siddarth et al., 2021). Each component score ranges from 0 to 3, with higher scores indicating worse sleep. The component scores were summed to produce a global score [range 0–21], with higher scores indicating poorer overall sleep quality. As per the scoring guidelines, if any component was missing, the global score was not calculated. The percentage of missing data on PSQI component scores ranged from 2.04% (*n* = 4) to 7.65% (*n* = 15). Of the 196 participants, 21 had missing data on one or more components and were excluded. As the distribution of PSQI global and component scores was not normal and was skewed, we dichotomized component scores based on previous studies (Abdulla et al., 2023; Siddarth et al., 2021) as follows:

- Global score: Good sleep quality (score ≤ 5) vs. bad sleep quality (score > 5)
- Sleep duration score: More than 7 h (score 0) vs. 7 h or less (score 1–3)
- Sleep disturbance score: Less disturbances (score 0–1) vs. more disturbances (score 2–3)
- Sleep latency score: 15 min or less (score 0) vs. more than 15 min (score 1–3)
- Daytime dysfunction score: Absence of dysfunction (score 0) vs. presence of dysfunction (score 1–3)
- Sleep efficiency score: More than 85% (score 0) vs. 85% or less (score 1–3)
- Sleep quality score: Good (score 0–1) vs. bad (score 2–3)
- Sleep medication score: Didn't use during the past month (score 0) vs. used during the past month (score 1–3)

#### Episodic memory

Participants completed three experimental measures of episodic memory. The first two, the Verbal Paired Associates Test and the Visual Paired Associates Test, were administered using The Many Brains Project's TestMyBrain cognitive testing platform (Singh et al., 2021). In the Verbal Paired Associates task, participants were presented with 25 word pairs (e.g., truck | lemon), one at a time for 3 s in a randomized order across participants. Participants were told to remember the pairs for a later memory test. Each pair was separated by a 0.5 s interstimulus interval (ISI). Following the encoding phase, participants completed the digital symbol matching task which lasted 1.5–2.5 min, depending on how quickly they completed all of the trials. Finally, participants were presented with one word from each of the studied word pairs, in a randomized order, and had 10 s to identify the word that had it had been paired with during the encoding phase from a list of four response options: one option was the correct response, one option was the correct response to another trial, one option was an incorrect response to another trial, and one option was a novel incorrect response. The Visual Paired Associates test was identical to the Verbal Paired Associates test, except participants viewed 24 image pairs which were unique examples of the same type of object or scene (e.g., two images of barns), and had to identify the correct match from five similar options. In this task, the incorrect options depicted a unique example of the same type of object or scene as the images in the original pair (e.g., barns). Between the encoding and recognition phases, participants completed the stop signal reaction time task, which took 1.5–2.5 min, depending on their speed. Accuracy, measured by the proportion of correctly answered trials, was the primary variable computed for both tests. Although the visual paired associates task requires participants to select a studied associate from among three visually similar foils and thus likely engages mnemonic discrimination processes, we conceptualized this task primarily as an index of associative memory and binding. In this task, lure discrimination is not isolated in the scoring, because participants make a 4-alternative forced choice with all options simultaneously available, performance can be supported by relative familiarity or elimination strategies, rather than by item-level pattern separation alone, and scoring does not isolate lure responses. Thus, we interpret the visual paired associates task primarily as an associative memory measure. The third test was the Mnemonic Similarity Task (MST) (Stark et al., 2019), which probes the ability to dissociate items that are similar to a previously presented item from items that are identical to a previously presented item, referred to as “pattern separation.” Pattern separation is thought to be a sensitive marker of hippocampal-dependent episodic memory, reflecting the ability to form distinct, non-overlapping representations of similar experiences. The task was completed while participants underwent fMRI. Participants saw 320 colored photographs of common objects, split into 4 blocks containing 80 trials each. On each trial, participants had to indicate whether the item was new (shown for the first time), old (an identical repeat of an item presented before), or similar (similar but not identical to an item previously presented). Within each block, there were 15 old items, 25 similar items, and 40 new items. Each item was presented one at a time for 3 s, followed by a 0.5 s ISI. Participants' responses (old, similar or new) were recorded during this 3.5 s window. The primary dependent variable was the lure discrimination index (LDI), calculated as the difference between the probability of responding similar to a similar item minus the probability of responding similar to a new item, which provides a response bias-corrected measure of pattern separation and episodic memory precision. Scores from the verbal and visual paired associates' tests and MST were converted to z-scores. The average z-score across the three task was taken to create an overall episodic memory score.

### Covariates

Age was measured in years. Sex assigned at birth and education were dichotomized (0 = male, 1 = female; 0 = up to some college, 1 = college degree or higher, respectively). Global cognitive function was evaluated using the Montreal Cognitive Assessment (MoCA) (Nasreddine et al., 2005). These covariates were selected to account for demographics and cognition.

### Statistical analysis

For descriptive analysis, we reported mean and standard deviation for continuous variables, median and interquartile range for ordinal variables, and frequencies and percentages for categorical variables. Associations between binary variables (sex, educational attainment, and PSQI global and component scores) were examined using chi-square tests. Associations between PSQI global and component scores (binary), episodic memory scores (continuous), and covariates (age, sex, educational attainment, and MoCA score) were examined using Pearson's correlation analysis. If a significant two-tailed correlation was found, independent *t*-tests were additionally conducted between binary and continuous variables.

Next, the associations between sleep and episodic memory were investigated using multivariate linear regressions controlling for age (mean centered), sex, educational attainment, and MoCA score. PSQI global score (Model 1) and the component scores (Model 2) were entered separately as independent variables in two regression models to test their main effects (Model 1a and 2a, respectively). Moreover, to examine the moderating effect of age, interaction terms between mean-centered age and the PSQI global score (Model 1b) and between mean-centered age and PSQI component scores (Model 2b) were added. We conducted multivariate linear regression analyses using continuous PSQI global and component scores separately. Although the PSQI global and component scores were dichotomized based on previous studies, the thresholds were arbitrary rather than clinically established. The results, which are not corrected for multiple comparisons, are presented in Supplementary Tables 3–6. As Bonferroni correction reduces power and increase a Type II error, it has been suggested that Pearson's *r* or Cohen's *d* be used to report effect sizes (Nakagawa, 2004). Cohen (1992) provided guidelines for interpreting effect sizes (Pearson's *r* = 0.10, 0.30, 0.50 and Cohen's *d* = 0.20, 0.50, 0.80 to interpret small, medium, and large effects) (Cohen, 1992). However, these guidelines may overestimate effect sizes in the field of gerontology and result in insufficient power to detect true effects (Brydges, 2019). Therefore, in this study, we interpreted our findings using the guidelines suggested by Brydges (2019) (Pearson's *r* = 0.10, 0.20, 0.30 and Cohen's *d* = 0.15, 0.40, 0.75 to interpret small, medium, and large effects). The statistical analysis was performed using STATA 19.5. A two-sided *p*-value of < 0.05 was considered statistically significant.

## Results

Among 196 participants in our study, we excluded those who were missing data on any variables of interest (*n* = 23). This led to a final sample size of 173 participants. Table 1 presents descriptive statistics of the study sample. The mean age was 65.30 (SD = 8.12). More than half of the sample were female (61.27%) and two-thirds of the sample were non-Hispanic White (67.05%). The majority of the sample obtained a college degree or above (75.14%) and the mean MoCA score was 26.93 (SD = 2.25). With regards to sleep disorders, thirteen percent of the sample reported ever having sleep apnea (13.29%) and 6.94% and 1.16% reported ever having insomnia and narcolepsy, respectively. About half of the sample reported poor overall sleep quality (46.24%). No participant in our sample reported currently using hypnotic or sedative drugs as classified under the Anatomical Therapeutic Chemical code by the World Health Organization (DDD Index, 2024).

**Table 1:** Sample characteristics (*N* = 173).

	**Estimates**
Age (years), *M* ± SD	65.30 ± 8.12
**Sex**, ***n*** **(%)**	
Male	67 (38.73%)
Female	106 (61.27%)
**Race/ethnicity**, ***n*** **(%)**	
Non-Hispanic White	116 (67.05%)
Non-Hispanic Black	12 (6.94%)
Hispanic	19 (10.98%)
Non-Hispanic other	21 (12.14%)
Unknown/preferred not to answer	5 (2.89%)
**Educational attainment**, ***n*** **(%)**	
Up to some college	43 (24.86%)
College degree or above	130 (75.14%)
MoCA score, *M* ± SD	26.93 ± 2.25
**Ever had sleep apnea**, ***n*** **(%)**	
No	150 (86.71%)
Yes	23 (13.29%)
**Ever had insomnia**, ***n*** **(%)**	
No	161 (93.06%)
Yes	12 (6.94%)
**Ever had narcolepsy**, ***n*** **(%)**	
No	171 (98.84%)
Yes	2 (1.16%)
**PSQI global score**, ***Mdn*** **(IQR)**	**5 (4–7)**
*Overall sleep quality based on PSQI global score, *n* (%)*	
Good sleep quality (PSQI Global ≤ 5)	93 (53.76%)
Poor sleep quality (PSQI Global > 5)	80 (46.24%)
**PSQI sleep duration score**, ***Mdn*** **(IQR)**	**1 (0–1)**
*Sleep duration based on PSQI component score, *n* (%)*	
More than 7 h	57 (32.95%)
7 h or less	116 (67.05%)
**PSQI sleep disturbance score**, ***Mdn*** **(IQR)**	**1 (1–2)**
*Sleep disturbance based on PSQI component score, *n* (%)*	
Less disturbances	119 (68.79%)
More disturbances	54 (31.21%)
**PSQI sleep latency score**, ***Mdn*** **(IQR)**	**1 (0–2)**
*Sleep latency based on PSQI component score, *n* (%)*	
15 min or less	59 (34.10%)
More than 15 min	114 (65.90%)
**PSQI daytime dysfunction score**, ***Mdn*** **(IQR)**	**1 (0–1)**
*Daytime dysfunction based on PSQI component score, *n* (%)*	
Absence of dysfunction	74 (42.77%)
Presence of dysfunction	99 (57.23%)
**PSQI sleep efficiency score**, ***Mdn*** **(IQR)**	**0 (0–1)**
*Sleep efficiency based on PSQI component score, *n* (%)*	
More than 85%	101 (58.38%)
85% or less	72 (41.62%)
**PSQI sleep quality score**, ***Mdn*** **(IQR)**	**1 (0–1)**
*Sleep quality based on PSQI component score, *n* (%)*	
Good quality	146 (84.39%)
Bad quality	27 (15.61%)
**PSQI sleep medication score**, ***Mdn*** **(IQR)**	**0 (0–0)**
*Sleep medication based on PSQI component score, *n* (%)*	
Didn't use during the past month	137 (79.19%)
Used during the past month	36 (20.81%)
**Episodic memory task performance**	
Visual paired associates memory (accuracy), *M* ± SD	0.58 ± 0.18
Verbal paired associates memory (accuracy), *M* ± SD	0.66 ± 0.23
MST LDI, *M* ±*SD*	0.47 ± 0.19

M, Mean; SD, Standard Deviation; Mdn, Median; IQR, Interquartile Range (25th percentile−75th percentile); MoCA, Montreal Cognitive Assessment; PSQI, Pittsburgh Sleep Quality Index; MST, Mnemonic Similarity Task; LDI, Lure Discrimination Index.

As a descriptive and exploratory step, we examined bivariate correlations among PSQI global and component scores, episodic memory scores, and covariates (Table 2). These analyses were conducted to show general association patterns rather than to draw inferential conclusions. Correlation analyses revealed that age was negatively associated with all of the episodic memory measures: Visual Paired Associates (*r* = −0.226), Verbal Paired Associates (*r* = −0.221), MST-LDI (*r* = −0.329) and the overall episodic memory score (*r* = −0.336), such that memory performance decreased with age. Age and MoCA were not correlated with any of the PSQI scores, but being female was positively correlated with greater sleep latency problems [*r* = 0.179; χ^2^(df = 1) = 5.54, *p* = 0.019], while having a higher level of education (college degree or above) was associated with fewer sleep problems, including a lower PSQI global score [*r* = −0.218; χ^2^(df = 1) = 8.20, =0.004], duration [*r* = −0.261; χ^2^(df = 1) = 11.77, *p* = 0.001], latency [*r* = −0.188; χ^2^(df = 1) = 6.12, *p* = 0.013], and efficiency scores [*r* = −0.193; χ^2^(df = 1) = 6.43, *p* = 0.011]. The chi-square test results can be seen in Supplementary Table 1.

**Table 2:** Correlations between sleep, episodic memory, and covariates (*N* = 173).

	**(1)**	**(2)**	**(3)**	**(4)**	**(5)**	**(6)**	**(7)**	**(8)**	**(9)**	**(10)**	**(11)**	**(12)**	**(13)**	**(14)**	**(15)**	**(16)**
(1)	1.000															
(2)	−0.137	1.000														
	(0.071)															
(3)	−0.030	−0.073	1.000													
	(0.697)	(0.341)														
(4)	−0.198	0.060	0.167	1.000												
	(0.009)	(0.432)	(0.028)													
(5)	−0.226	0.077	0.029	0.338	1.000											
	(0.003)	(0.311)	(0.700)	(0.000)												
(6)	−0.221	0.100	0.257	0.394	0.387	1.000										
	(0.004)	(0.192)	(0.001)	(0.000)	(0.000)											
(7)	−0.329	0.094	0.092	0.421	0.433	0.350	1.000									
	(0.000)	(0.218)	(0.229)	(0.000)	(0.000)	(0.000)										
(8)	−0.336	0.118	0.165	0.499	0.783	0.754	0.774	1.000								
	(0.000)	(0.124)	(0.030)	(0.000)	(0.000)	(0.000)	(0.000)									
(9)	−0.032	0.047	−0.218	−0.039	−0.037	0.054	−0.096	−0.034	1.000							
	(0.680)	(0.538)	(0.004)	(0.615)	(0.627)	(0.482)	(0.209)	(0.655)								
(10)	0.061	−0.078	−0.261	−0.038	−0.081	0.014	−0.099	−0.072	0.305	1.000						
	(0.426)	(0.310)	(0.001)	(0.618)	(0.289)	(0.853)	(0.194)	(0.349)	(0.000)							
(11)	−0.111	−0.053	−0.046	−0.007	0.056	0.044	0.044	0.062	0.401	0.127	1.000					
	(0.145)	(0.485)	(0.552)	(0.927)	(0.468)	(0.567)	(0.564)	(0.417)	(0.000)	(0.095)						
(12)	−0.052	0.179	−0.188	−0.093	−0.063	0.009	−0.129	−0.079	0.447	0.015	0.169	1.000				
	(0.500)	(0.018)	(0.013)	(0.224)	(0.411)	(0.903)	(0.090)	(0.300)	(0.000)	(0.849)	(0.026)					
(13)	−0.004	0.080	−0.119	−0.022	−0.027	0.062	−0.093	−0.025	0.333	0.040	0.179	0.216	1.000			
	(0.959)	(0.295)	(0.120)	(0.778)	(0.728)	(0.420)	(0.224)	(0.745)	(0.000)	(0.599)	(0.018)	(0.004)				
(14)	0.009	0.045	−0.193	0.000	−0.068	−0.092	−0.137	−0.129	0.463	0.292	0.013	0.162	0.066	1.000		
	(0.904)	(0.553)	(0.011)	(1.000)	(0.372)	(0.228)	(0.072)	(0.090)	(0.000)	(0.000)	(0.862)	(0.033)	(0.386)			
(15)	−0.061	0.048	−0.084	−0.029	0.064	0.035	−0.106	−0.004	0.368	0.234	0.226	0.108	0.146	0.186	1.000	
	(0.424)	(0.534)	(0.270)	(0.702)	(0.403)	(0.648)	(0.165)	(0.960)	(0.000)	(0.002)	(0.003)	(0.158)	(0.055)	(0.014)		
(16)	0.030	−0.031	0.064	−0.029	0.077	0.131	0.072	0.122	0.353	−0.065	0.085	0.128	0.127	0.145	0.093	1.000
	(0.693)	(0.686)	(0.402)	(0.709)	(0.312)	(0.086)	(0.345)	(0.111)	(0.000)	(0.397)	(0.267)	(0.092)	(0.097)	(0.057)	(0.221)	

Estimates are Pearson correlations and *p*-values are in parentheses.

(1) Age, (2) Sex, (3) Educational attainment, (4) MOCA score, (5) Visual paired associates memory (z-score), (6) Verbal paired associates memory (z-score), (7) MST LDI (z-score), (8) Overall memory score, (9) PSQI global score, (10) PSQI sleep duration, (11) PSQI sleep disturbance, (12) PSQI sleep latency, (13) PSQI day dysfunction, (14) PSQI sleep efficiency, (15) PSQI sleep quality, (16) PSQI sleep medication.

MoCA, Montreal Cognitive Assessment; PSQI, Pittsburgh Sleep Quality Index; MST, Mnemonic Similarity Task; LDI, Lure Discrimination Index.

**Figure 1:**
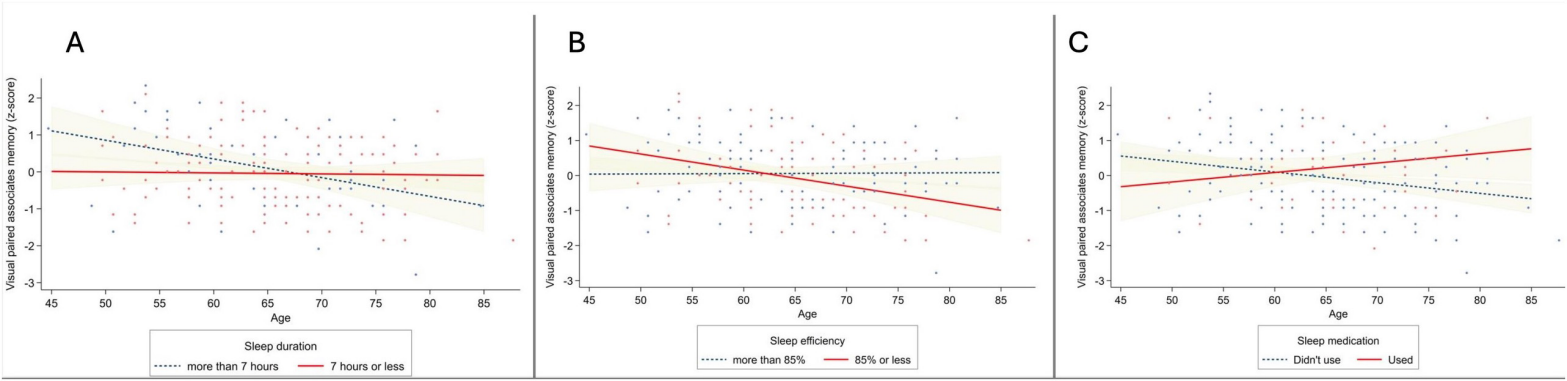
Associations between age and visual paired associates memory performance as a function of **(A)** sleep duration, with navy dots representing observed visual paired associates memory datapoints of participants with sleep duration of more than 7 h and red dots represent those with 7 h or less; **(B)** sleep efficiency, with navy dots representing observed visual paired associates memory datapoints of participants with sleep efficiency of more than 85% and red dots representing those with 85% or less; and **(C)** sleep medication use, with navy dots representing participants who did not use sleep medication during the past month and red dots represent those who did. Shaded areas represent the 95% confidence interval. Graphs displayed after linear regression analysis with age mean-centered and covariates controlled.

Tables 3–6 show the results of linear regression analyses examining the associations between PSQI global and component scores and each memory measure. For visual paired associates memory (Table 3), significant associations were observed between age and sleep duration (β = 0.319, *p* = 0.018, partial *r* = 0.189, Cohen's *d* = 0.386), age and sleep efficiency (β = −0.226, *p* = 0.019, partial *r* = 0.187, Cohen's *d* = 0.382), and age and sleep medication (β = 0.191, *p* = 0.023, partial *r* = 0.182, Cohen's *d* = 0.369). With increasing age, participants who slept more than 7 h (Figure 1A) or had 85% or less sleep efficiency (Figure 1B) showed downward trends in visual paired associates memory performance, whereas those who used sleep medication during the past month showed upward trends (Figure 1C). The interactions between age and sleep efficiency (β = −0.195, *p* = 0.043, partial *r* = 0.162, Cohen's *d* = 0.329) and age and sleep medication (β = 0.176, *p* = 0.036, partial *r* = 0.168, Cohen's *d* = 0.341) remained significant in linear regression analyses using continuous PSQI component scores (Supplementary Table 3).

**Table 3:** Linear regression of associations between sleep and visual paired associates memory (*N* = 173).

	**Model 1a**	**Model 1b**	**Model 2a**	**Model 2b**
	β **(SE)**	β **(SE)**	β **(SE)**	β **(SE)**
PSQI global score (ref. good sleep quality)	−0.039	−0.040		
	(0.144)	(0.144)		
Age (mean-centered)	−0.162^*^	−0.222^*^	−0.152^*^	−0.450^**^
	(0.009)	(0.011)	(0.009)	(0.020)
PSQI global score × age		0.097		
		(0.018)		
Sex (ref. male)	0.036	0.025	0.049	0.023
	(0.146)	(0.147)	(0.150)	(0.148)
Educational attainment (ref. up to some college)	−0.033	−0.042	−0.073	−0.098
	(0.169)	(0.170)	(0.175)	(0.174)
MoCA score	0.307^***^	0.315^***^	0.312^***^	0.301^***^
	(0.032)	(0.033)	(0.033)	(0.032)
PSQI sleep duration (ref. more than 7 h)			−0.066	−0.067
			(0.166)	(0.162)
PSQI sleep disturbance (ref. less disturbances)			0.042	0.059
			(0.161)	(0.171)
PSQI sleep latency (ref. 15 min or less)			−0.071	−0.053
			(0.160)	(0.160)
PSQI daytime dysfunction (ref. no)			−0.043	−0.045
			(0.149)	(0.150)
PSQI sleep efficiency (ref. more than 85%)			−0.081	−0.068
			(0.155)	(0.153)
PSQI sleep quality (ref. good quality)			0.080	0.014
			(0.207)	(0.218)
PSQI sleep medication (ref. no)			0.108	0.114
			(0.179)	(0.178)
PSQI sleep duration × age				0.319^*^
				(0.020)
PSQI sleep disturbance × age				0.138
				(0.020)
PSQI sleep latency × age				−0.007
				(0.019)
PSQI daytime dysfunction × age				0.091
				(0.018)
PSQI sleep efficiency × age				−0.226^*^
				(0.020)
PSQI sleep quality × age				−0.127
				(0.027)
PSQI sleep medication × age				0.191^*^
				(0.025)

^*^p < 0.05, ^**^p < 0.01, ^***^p < 0.001.

β, standardized beta coefficient; SE, standard errors; ref., reference group; MoCA, Montreal Cognitive Assessment; PSQI, Pittsburgh Sleep Quality Index.

PSQI global score (Model 1) and the component scores (Model 2) were entered separately as independent variables in two regression models to test their main effects (Model 1a and 2a, respectively). Moreover, to examine the moderating effect of age, interaction terms between mean-centered age and the PSQI global score (Model 1b) and between mean-centered age and PSQI component scores (Model 2b) were added.

**Figure 2:**
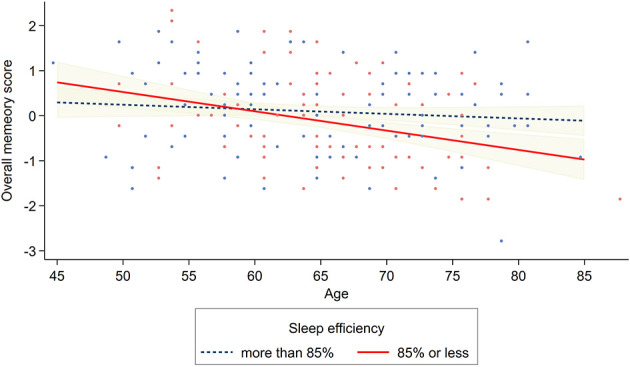
Association between age and overall episodic memory as a function of PSQI sleep efficiency. Participants with ≤85% sleep efficiency showed a decline in overall memory performance with increasing age. Note: Shaded area represents the 95% confidence interval. Navy dots represent observed overall memory datapoints of participants with sleep efficiency of more than 85%. Red dots represent those with 85% or less. Graph displayed after linear regression analysis with age mean-centered and covariates controlled.

For verbal paired associates memory and MST LDI (Tables 4, 5), there was a significant main effect of sleep medication, indicating that the use of sleep medications was associated with better verbal paired associates memory (β = 0.147, *p* = 0.038, partial *r* = 0.162, Cohen's *d* = 0.329) and pattern separation (β = 0.140, *p* = 0.042, partial *r* = 0.159, Cohen's *d* = 0.323). In addition, when PSQI component scores were treated as continuous variables in regression analyses, there was a significant interaction between age and sleep efficiency (β = −0.207, *p* = 0.023, partial *r* = 0.183, Cohen's *d* = 0.371) on MST LDI (Supplementary Table 5). With respect to overall memory score (Table 6), there was a significant main effect of sleep efficiency (β = −0.144, *p* = 0.037, partial *r* = 0.163, Cohen's *d* = 0.331) and medication (β = 0.171, *p* = 0.010, partial *r* = 0.202, Cohen's *d* = 0.412), such that higher sleep efficiency (>85%) and the use of sleep medication was associated with better overall memory score. Furthermore, there was a significant association between age and sleep efficiency (β = −0.201, *p* = 0.019, partial *r* = 0.187, Cohen's *d* = 0.381) on the overall memory score, indicating that participants with 85% or less sleep efficiency showed downward trends in the overall memory score with increasing age (see Figure 2). The interaction between age and sleep efficiency was significant in linear regression analyses using continuous PSQI component scores (β = −0.184, *p* = 0.032, partial *r* = 0.171, Cohen's *d* = 0.348), whereas that between age and sleep medication was not (Supplementary Table 6).

**Table 4:** Linear regression of associations between sleep and verbal paired associates memory (*N* = 173).

	**Model 1a**	**Model 1b**	**Model 2a**	**Model 2b**
	β **(SE)**	β **(SE)**	β **(SE)**	β **(SE)**
PSQI global score (ref. good sleep quality)	0.108	0.109		
	(0.140)	(0.140)		
Age (mean-centered)	−0.136	−0.072	−0.145^*^	0.047
	(0.009)	(0.011)	(0.009)	(0.020)
PSQI global score × age		−0.103		
		(0.018)		
Sex (ref. male)	0.073	0.084	0.083	0.097
	(0.142)	(0.143)	(0.145)	(0.148)
Educational attainment (ref. up to some college)	0.227^**^	0.237^**^	0.222^**^	0.224^**^
	(0.164)	(0.165)	(0.169)	(0.174)
MoCA score	0.329^***^	0.320^***^	0.339^***^	0.330^***^
	(0.031)	(0.032)	(0.031)	(0.032)
PSQI sleep duration (ref. more than 7 h)			0.141	0.146
			(0.160)	(0.162)
PSQI sleep disturbance (ref. less disturbances)			−0.006	−0.025
			(0.156)	(0.171)
PSQI sleep latency (ref. 15 min or less)			0.046	0.053
			(0.155)	(0.160)
PSQI daytime dysfunction (ref. no)			0.061	0.058
			(0.143)	(0.151)
PSQI sleep efficiency (ref. more than 85%)			−0.129	−0.120
			(0.150)	(0.153)
PSQI sleep quality (ref. good quality)			0.016	0.008
			(0.200)	(0.218)
PSQI sleep medication (ref. no)			0.147^*^	0.156^*^
			(0.173)	(0.179)
PSQI sleep duration × age				0.024
				(0.020)
PSQI sleep disturbance × age				−0.008
				(0.020)
PSQI sleep latency × age				−0.183
				(0.019)
PSQI daytime dysfunction × age				−0.021
				(0.018)
PSQI sleep efficiency × age				−0.086
				(0.020)
PSQI sleep quality × age				−0.010
				(0.027)
PSQI sleep medication × age				0.008
				(0.025)

^*^p < 0.05, ^**^p < 0.01, ^***^p < 0.001.

β, standardized beta coefficient; SE, standard errors; ref., reference group; MoCA, Montreal Cognitive Assessment; PSQI, Pittsburgh Sleep Quality Index.

PSQI global score (Model 1) and the component scores (Model 2) were entered separately as independent variables in two regression models to test their main effects (Model 1a and 2a, respectively). Moreover, to examine the moderating effect of age, interaction terms between mean-centered age and the PSQI global score (Model 1b) and between mean-centered age and PSQI component scores (Model 2b) were added.

**Table 5:** Linear regression of associations between sleep and MST LDI (*N* = 173).

	**Model 1a**	**Model 1b**	**Model 2a**	**Model 2b**
	β **(SE)**	β **(SE)**	β **(SE)**	β **(SE)**
PSQI global score (ref. good sleep quality)	−0.090	−0.090		
	(0.138)	(0.138)		
Age (mean-centered)	−0.254^***^	−0.198^*^	−0.255^***^	−0.189
	(0.009)	(0.011)	(0.009)	(0.020)
PSQI global score × age		−0.090		
		(0.017)		
Sex (ref. male)	0.042	0.052	0.078	0.081
	(0.140)	(0.141)	(0.141)	(0.144)
Educational attainment (ref. up to some college)	0.007	0.016	−0.041	−0.043
	(0.162)	(0.163)	(0.166)	(0.169)
MoCA score	0.363^***^	0.356^***^	0.361^***^	0.357^***^
	(0.031)	(0.031)	(0.031)	(0.031)
PSQI sleep duration (ref. more than 7 h)			−0.010	−0.006
			(0.157)	(0.158)
PSQI sleep disturbance (ref. less disturbances)			0.067	0.085
			(0.152)	(0.166)
PSQI sleep latency (ref. 15 min or less)			−0.112	−0.103
			(0.151)	(0.155)
PSQI daytime dysfunction (ref. no)			−0.080	−0.087
			(0.140)	(0.146)
PSQI sleep efficiency (ref. more than 85%)			−0.123	−0.121
			(0.146)	(0.148)
PSQI sleep quality (ref. good quality)			−0.097	−0.140
			(0.195)	(0.212)
PSQI sleep medication (ref. no)			0.140^*^	0.143^*^
			(0.169)	(0.174)
PSQI sleep duration × age				0.119
				(0.020)
PSQI sleep disturbance × age				0.010
				(0.020)
PSQI sleep latency × Age				−0.042
				(0.018)
PSQI daytime dysfunction × age				0.039
				(0.018)
PSQI sleep efficiency × age				−0.154
				(0.019)
PSQI sleep quality × age				−0.118
				(0.026)
PSQI sleep medication × age				0.087
				(0.024)

^*^p < 0.05, ^***^p < 0.001.

β, standardized beta coefficient; SE, standard errors; ref., reference group; MoCA, Montreal Cognitive Assessment; PSQI, Pittsburgh Sleep Quality Index; MST, Mnemonic Similarity Task; LDI, Lure Discrimination Index.

PSQI global score (Model 1) and the component scores (Model 2) were entered separately as independent variables in two regression models to test their main effects (Model 1a and 2a, respectively). Moreover, to examine the moderating effect of age, interaction terms between mean-centered age and the PSQI global score (Model 1b) and between mean-centered age and PSQI component scores (Model 2b) were added.

**Table 6:** Linear regression of associations between sleep and overall memory score (*N* = 173).

	**Model 1a**	**Model 1b**	**Model 2a**	**Model 2b**
	β **(SE)**	β **(SE)**	β **(SE)**	β **(SE)**
PSQI global score (ref. good sleep quality)	−0.009	−0.009		
	(0.101)	(0.101)		
Age (mean-centered)	−0.239^***^	−0.212^*^	−0.239^***^	−0.254
	(0.006)	(0.008)	(0.006)	(0.014)
PSQI global score × age		−0.043		
		(0.013)		
Sex (ref. male)	0.066	0.070	0.091	0.088
	(0.102)	(0.103)	(0.103)	(0.103)
Educational attainment (ref. up to some college)	0.088	0.092	0.048	0.037
	(0.118)	(0.119)	(0.121)	(0.121)
MoCA score	0.433^***^	0.429^***^	0.438^***^	0.428^***^
	(0.023)	(0.023)	(0.022)	(0.023)
PSQI sleep duration (ref. more than 7 h)			0.029	0.033
			(0.114)	(0.113)
PSQI sleep disturbance (ref. less disturbances)			0.044	0.051
			(0.111)	(0.119)
PSQI sleep latency (ref. 15 min or less)			−0.059	−0.044
			(0.110)	(0.111)
PSQI daytime dysfunction (ref. no)			−0.026	−0.031
			(0.102)	(0.105)
PSQI sleep efficiency (ref. more than 85%)			−0.144^*^	−0.134
			(0.106)	(0.106)
PSQI sleep quality (ref. good quality)			−0.002	−0.052
			(0.142)	(0.152)
PSQI sleep medication (ref. no)			0.171^**^	0.179^**^
			(0.123)	(0.125)
PSQI sleep duration × age				0.198
				(0.014)
PSQI sleep disturbance × age				0.060
				(0.014)
PSQI sleep latency × age				−0.101
				(0.013)
PSQI daytime dysfunction × age				0.012
				(0.013)
PSQI sleep efficiency × age				−0.201^*^
				(0.014)
PSQI sleep quality × age				−0.110
				(0.019)
PSQI sleep medication × age				0.123
				(0.018)

^*^p < 0.05, ^**^p < 0.01, ^***^p < 0.001.

β, standardized beta coefficient; SE, standard errors; ref., reference group; MoCA, Montreal Cognitive Assessment; PSQI, Pittsburgh Sleep Quality Index.

PSQI global score (Model 1) and the component scores (Model 2) were entered separately as independent variables in two regression models to test their main effects (Model 1a and 2a, respectively). Moreover, to examine the moderating effect of age, interaction terms between mean-centered age and the PSQI global score (Model 1b) and between mean-centered age and PSQI component scores (Model 2b) were added.

## Discussion

This study examined associations between individual and global indices of self-reported sleep quality—analyzed both continuously and dichotomously—and age-related variation across several measures of episodic memory. We observed a significant main effect of sleep medication use on 3 of the 4 episodic memory measures (verbal, pattern separation (MST-LDI), and overall episodic memory), such that greater medication use was associated with better memory. Individuals with high sleep efficiency (>85%) also showed superior overall episodic memory. Age significantly interacted with sleep medication, sleep efficiency, and sleep duration in predicting visual memory, as well as with sleep efficiency in predicting overall memory. When component scores were treated as continuous variables, the main effects of sleep medication on pattern separation (MST-LDI) and overall memory, as well as the main effect of efficiency on overall memory, were no longer significant, nor was the age-by-duration interaction on visual memory. Due to the lack of consistent findings between sleep duration and episodic memory measures, we believe an objective measure of duration may be better suited to explain this difference in effect.

These findings follow the expected pattern of results whereby worse episodic memory is associated with overall poorer sleep quality, as demonstrated by prior research (Ma et al., 2020; Overton et al., 2024; Saint Martin et al., 2012). Recent work exploring different aspects of subjective sleep has also reported that worse sleep efficiency is associated with impairments in working memory in older adults (Miyata et al., 2013). Our study extends this finding to episodic memory. Previous research also indicates that the effects of sleep medications differ based on the mechanisms of action (Beracochea, 2006), and may be specific to drugs that target the GABAergic system (Zhang et al., 2020). Mechanistically, better sleep efficiency and the use of sleep medications may benefit memory-related processes through the neural correlates of sleep. According to the two-stage model, spindles, slow oscillations, and ripples during SWS synchronize hippocampal–neocortical communication to facilitate memory consolidation, while subsequent REM sleep promotes synaptic plasticity that supports future memory formation (Diekelmann and Born, 2010). Therefore, efficient sleep may strengthen these processes and improve performance on episodic memory tasks. Additionally, although most sleep medications, specifically benzodiazepines, zolpidem, and zopiclone are known to suppress SWS (Lancel, 1999), other GABAergic and non-GABAergic agents, including 5HT2A antagonists, can enhance SWS (Dijk, 2010). Although we did not have detailed information on the specific sleep medications used by participants, we excluded those taking GABAergic medications, which may help to explain the observed effects of sleep medication and better episodic memory performance. Further research using longitudinal study models may help to clarify the direction of the relationship between sleep medication and measures of episodic memory.

## Limitations

Several important limitations of this study should be noted. First, only about 20% of participants reported taking sleep medications, which limits the generalizability of our findings to clinical populations with more severe sleep disorders such as insomnia, obstructive sleep apnea, or circadian rhythm disorders. Moreover, greater detail on specific sleep medications would help clarify their mechanisms of action and potential age-specific effects on episodic memory. Second, sleep quality was assessed using a self-report questionnaire. Although this measure is widely used to capture subjective sleep experience (Brewster et al., 2015), responses may be influenced by recall bias, social desirability, and individual differences in how items are interpreted. In addition, retrospective reports of sleep may themselves be affected by memory performance—such that poorer memory could distort recall of one's sleep quality. Future work incorporating both subjective and objective assessments of sleep could help address these concerns. Third, we used traditional linear regression models because they allow for modeling interactions and produce interpretable results; however, they assume linear relationships and may fail to capture more complex or non-linear effects (Clark and Hou, 2021). Future studies could consider alternative analytic approaches such as LASSO (least absolute shrinkage and selection operator) regression or Random Forests to better model non-linear associations and improve variable selection (Clark and Hou, 2021). Fourth, while we could not directly examine relationships between subjective sleep measures and APOE4 carrier status or Alzheimer's disease (AD) pathology, these associations remain conceptually important for understanding disease prevention and treatment. Sleep efficiency has been linked to AD pathology—for example, preclinical AD patients with greater amyloid deposition show reduced sleep efficiency (Abdulla et al., 2023). Previous research also found age-dependent associations between sleep duration and dementia, with short duration in younger adults and long duration in older adults linked to higher dementia risk (Xiong et al., 2024). Additionally, an existing protocol for systematic reviews examining objective sleep measures as indicators of dementia (Antonioni et al., 2025), and ongoing clinical trials focused on how obstructive sleep apnea influences amyloid and tau burden (Malhotra, 2024), further underscore the relevance of sleep to AD pathophysiology. In addition, prior research suggests that sleep medication use may mitigate the risk of MCI among APOE4 carriers (Siddarth et al., 2021). Although APOE4 status was not collected in this study, approximately 25% of the population carry one copy of the allele, a major genetic risk factor for AD. According to the model proposed by Jack et al. (2010), pathological changes in AD emerge well before the onset of cognitive symptoms—most notably in episodic memory—and the duration of this preclinical period likely varies across individuals. Fifth, to be consistent with previous thresholds used in sleep research, and to maintain consistency across sleep components, we retained the dichotomized PSQI components in the analyses. However, when raw continuous sleep duration, sleep latency, and sleep efficiency values were examined additionally, the associations with episodic memory were attenuated and no longer statistically significant. It is possible thus that dichotomization masked important within-component variation, and that sleep–memory associations may not be strictly linear across the underlying continuous measures. Finally, when we applied a false discovery rate (FDR) correction, no associations survived. Even though we followed recommendations to prioritize effect sizes over dichotomous *p*-value thresholds, interpretation should be made with caution. Despite these limitations, however, taken together, our results suggest that sleep efficiency and medication use may play key roles in supporting episodic memory across age, emphasizing the need for longitudinal and multimodal studies to clarify causal pathways and their relevance for AD prevention.

## Data Availability

The raw data supporting the conclusions of this article will be made available by the authors, without undue reservation.

## References

[B1] Abdulla N. K. Obaid R. R. Qureshi M. N. Asraiti A. A. Janahi M. A. Abu Qiyas S. J. . (2023). Relationship between hedonic hunger and subjectively assessed sleep quality and perceived stress among university students: a cross-sectional study. Heliyon 9:e14987. doi: 10.1016/j.heliyon.2023.e14987 37089280 PMC10114148

[B2] Aly M. Moscovitch M. (2010). The effects of sleep on episodic memory in older and younger adults. Memory 18, 327–334. doi: 10.1080/09658211003601548 20182945

[B3] Antonioni A. Della Valle A. Leitner C. Raho E. M. Cesnik E. Capone J. G. . (2025). Sleep disturbances across dementias and cognitive decline: study protocol for a systematic review and network meta-analysis of polysomnographic findings. J. Clin. Med. 14:7437. doi: 10.3390/jcm14207437 41156316 PMC12565185

[B4] Beracochea D. (2006). Anterograde and retrograde effects of benzodiazepines on memory. The Sci. World J. 6:618319. doi: 10.1100/tsw.2006.243 17115086 PMC5917174

[B5] Bonnet M. H. (1989). The effect of sleep fragmentation on sleep and performance in younger and older subjects. Neurobiol. Aging 10, 21–25. doi: 10.1016/S0197-4580(89)80006-5 2755554

[B6] Brewster G. S. Varrasse M. Rowe M. (2015). Sleep and cognition in community-dwelling older adults: a review of literature. Healthcare 3, 1243–1270. doi: 10.3390/healthcare3041243 27066397 PMC4822499

[B7] Brydges C. R. (2019). Effect size guidelines, sample size calculations, and statistical power in gerontology. Innov. Aging 3:igz036. doi: 10.1093/geroni/igz036 31528719 PMC6736231

[B8] Buschke H. (1973). Selective reminding for analysis of memory and learning. J. Verbal Learn. Verbal Behav. 12, 543–550. doi: 10.1016/S0022-5371(73)80034-9

[B9] Buysse D. J. Reynolds C. F. Monk T. H. Berman S. R. Kupfer D. J. (1989). The pittsburgh sleep quality index: a new instrument for psychiatric practice and research. Psychiatry Res. 28, 193–213. doi: 10.1016/0165-1781(89)90047-4 2748771

[B10] Clark R. R. S. Hou J. (2021). Three machine learning algorithms and their utility in exploring risk factors associated with primary cesarean section in low-risk women: a methods paper. Res. Nurs. Health 44, 559–570. doi: 10.1002/nur.22122 33651381 PMC8068617

[B11] Cohen D. E. Kim H. Levine A. Devanand D. P. Lee S. Goldberg T. E. (2024). Effects of age on the relationship between sleep quality and cognitive performance: findings from the Human Connectome Project-Aging cohort. Int. Psychogeriatr. 36, 1171–1181. doi: 10.1017/S1041610223000911 38047419 PMC11147958

[B12] Cohen J. (1992). A power primer. Psychol. Bull. 112, 155–159. doi: 10.1037/0033-2909.112.1.155 19565683

[B13] Craik F. I. M. (1994). Memory changes in normal aging. Curr. Dir. Psychol. Sci. 3, 155–158. doi: 10.1111/1467-8721.ep10770653

[B14] DDD Index (2024). Available online at: https://atcddd.fhi.no/atc_ddd_index/?code=N05C (Accessed November 7, 2025).

[B15] Diekelmann S. Born J. (2010). The memory function of sleep. Nat. Rev. Neurosci. 11, 114–126. doi: 10.1038/nrn2762 20046194

[B16] Dijk D.-J. (2010). Slow-wave sleep deficiency and enhancement: implications for insomnia and its management. World J. Biol. Psychiatry 11, 22–28. doi: 10.3109/15622971003637645 20509829

[B17] Jack C. R. Jr Knopman D. S. Jagust W. J. Shaw L. M. Aisen P. S. Weiner M. W. . (2010). Hypothetical model of dynamic biomarkers of the Alzheimer's pathological cascade. Lancet Neurol. 9, 119–128. doi: 10.1016/S1474-4422(09)70299-6 20083042 PMC2819840

[B18] Lancel M. (1999). Role of GABAA receptors in the regulation of sleep: initial sleep responses to peripherally administered modulators and agonists. Sleep 22, 33–42. doi: 10.1093/sleep/22.1.33 9989364

[B19] Ma Y. Liang L. Zheng F. Shi L. Zhong B. Xie W. (2020). Association between sleep duration and cognitive decline. JAMA Netw. Open 3:e2013573. doi: 10.1001/jamanetworkopen.2020.13573 32955572 PMC7506513

[B20] Malhotra A. (2024). Is Obstructive Sleep Apnea Important in the Development of Alzheimer's Disease? Available online at: https://clinicaltrials.gov/study/NCT05094271 (Accessed November 7, 2025).

[B21] Mander B. A. Winer J. R. Walker M. P. (2017). Sleep and human aging. Neuron 94, 19–36. doi: 10.1016/j.neuron.2017.02.004 28384471 PMC5810920

[B22] Miyata S. Noda A. Iwamoto K. Kawano N. Okuda M. Ozaki N. (2013). Poor sleep quality impairs cognitive performance in older adults. J. Sleep Res. 22, 535–541. doi: 10.1111/jsr.12054 23560612

[B23] Nakagawa S. (2004). A farewell to Bonferroni: the problems of low statistical power and publication bias. Behav. Ecol. 15, 1044–1045. doi: 10.1093/beheco/arh107

[B24] Nasreddine Z. S. Phillips N. A. Bédirian V. Charbonneau S. Whitehead V. Collin I. . (2005). The montreal cognitive assessment, MoCA: a brief screening tool for mild cognitive impairment. J. Am. Geriatr. Soc. 53, 695–699. doi: 10.1111/j.1532-5415.2005.53221.x 15817019

[B25] Ohayon M. M. Carskadon M. A. Guilleminault C. Vitiello M. V. (2004). Meta-analysis of quantitative sleep parameters from childhood to old age in healthy individuals: developing normative sleep values across the human lifespan. Sleep 27, 1255–1273. doi: 10.1093/sleep/27.7.1255 15586779

[B26] Overton M. Skoog J. Laukka E. J. Bodin T. H. Mattsson A. D. Sjöberg L. . (2024). Sleep disturbances and change in multiple cognitive domains among older adults: a multicenter study of five Nordic cohorts. Sleep 47:zsad244. doi: 10.1093/sleep/zsad244 37708350 PMC10925948

[B27] Ravyts S. G. Dzierzewski J. M. (2024). Sleep and healthy aging: a systematic review and path forward. Clin. Gerontol. 47, 367–379. doi: 10.1080/07317115.2022.2064789 35445642 PMC9585152

[B28] Saint Martin M. Sforza E. Barthélémy J. C. Thomas-Anterion C. Roche F. (2012). Does subjective sleep affect cognitive function in healthy elderly subjects? The Proof cohort. Sleep Med. 13, 1146–1152. doi: 10.1016/j.sleep.2012.06.021 22875008

[B29] Siddarth P. Thana-Udom K. Ojha R. Merrill D. Dzierzewski J. M. Miller K. . (2021). Sleep quality, neurocognitive performance, and memory self-appraisal in middle-aged and older adults with memory complaints. Int. Psychogeriatr. 33, 703–713. doi: 10.1017/S1041610220003324 32985406 PMC8004546

[B30] Singh S. Strong R. W. Jung L. Li F. H. Grinspoon L. Scheuer L. S. . (2021). The TestMyBrain digital neuropsychology toolkit: development and psychometric characteristics. J. Clin. Exp. Neuropsychol. 43, 786–795. doi: 10.1080/13803395.2021.2002269 34907842 PMC8922997

[B31] Stark S. M. Kirwan C. B. Stark C. E. L. (2019). Mnemonic similarity task: a tool for assessing hippocampal integrity. Trends Cogn. Sci. 23, 938–951. doi: 10.1016/j.tics.2019.08.003 31597601 PMC6991464

[B32] Xiong Y. Tvedt J. Åkerstedt T. Cadar D. Wang H.-X. (2024). Impact of sleep duration and sleep disturbances on the incidence of dementia and Alzheimer's disease: a 10-year follow-up study. Psychiatry Res. 333:115760. doi: 10.1016/j.psychres.2024.115760 38301285

[B33] Zhang J. Yetton B. Whitehurst L. N. Naji M. Mednick S. C. (2020). The effect of zolpidem on memory consolidation over a night of sleep. Sleep 43:zsaa084. doi: 10.1093/sleep/zsaa084 32330272 PMC8064806

