# Supplementary material for: Divergent effects of sleep efficiency and sleep medication on episodic memory in mid to late life
Source: Front Sleep. 2026 Jan 12;4:1691035. doi: 10.3389/frsle.2025.1691035 (PMC12832419; doi:10.3389/frsle.2025.1691035)
Supplement: Supplementary file 1 [file Table_1.docx]

| **Supplemental Table 1. Chi-square tests of association among binary variables (*N* = 173)** | | | | |
| --- | --- | --- | --- | --- |
| Variable 1 | Variable 2 | *χ^2^* | *df* | p-value |
| Sex | Educational attainment | 0.92 | 1 | 0.338 |
| Sex | PSQI global score | 0.39 | 1 | 0.535 |
| Sex | PSQI sleep duration | 1.04 | 1 | 0.307 |
| Sex | PSQI sleep disturbance | 0.49 | 1 | 0.482 |
| Sex | PSQI sleep latency | 5.54 | 1 | **0.019** |
| Sex | PSQI daytime dysfunction | 1.11 | 1 | 0.292 |
| Sex | PSQI sleep efficiency | 0.36 | 1 | 0.551 |
| Sex | PSQI sleep quality | 0.39 | 1 | 0.531 |
| Sex | PSQI sleep medication | 0.17 | 1 | 0.684 |
| Educational attainment | PSQI global score | 8.20 | 1 | **0.004** |
| Educational attainment | PSQI sleep duration | 11.77 | 1 | **0.001** |
| Educational attainment | PSQI sleep disturbance | 0.36 | 1 | 0.549 |
| Educational attainment | PSQI sleep latency | 6.12 | 1 | **0.013** |
| Educational attainment | PSQI daytime dysfunction | 2.44 | 1 | 0.118 |
| Educational attainment | PSQI sleep efficiency | 6.43 | 1 | **0.011** |
| Educational attainment | PSQI sleep quality | 1.23 | 1 | 0.267 |
| Educational attainment | PSQI sleep medication | 0.71 | 1 | 0.399 |
| PSQI global score | PSQI sleep duration | 16.08 | 1 | **0.000** |
| PSQI global score | PSQI sleep disturbance | 27.82 | 1 | **0.000** |
| PSQI global score | PSQI sleep latency | 34.59 | 1 | **0.000** |
| PSQI global score | PSQI daytime dysfunction | 19.21 | 1 | **0.000** |
| PSQI global score | PSQI sleep efficiency | 37.16 | 1 | **0.000** |
| PSQI global score | PSQI sleep quality | 23.41 | 1 | **0.000** |
| PSQI global score | PSQI sleep medication | 21.53 | 1 | **0.000** |
| PSQI sleep duration | PSQI sleep disturbance | 2.80 | 1 | 0.094 |
| PSQI sleep duration | PSQI sleep latency | 0.04 | 1 | 0.848 |
| PSQI sleep duration | PSQI daytime dysfunction | 0.28 | 1 | 0.597 |
| PSQI sleep duration | PSQI sleep efficiency | 14.80 | 1 | **0.000** |
| PSQI sleep duration | PSQI sleep quality | 9.45 | 1 | **0.002** |
| PSQI sleep duration | PSQI sleep medication | 0.73 | 1 | 0.394 |
| PSQI sleep disturbance | PSQI sleep latency | 4.93 | 1 | **0.026** |
| PSQI sleep disturbance | PSQI daytime dysfunction | 5.54 | 1 | **0.019** |
| PSQI sleep disturbance | PSQI sleep efficiency | 0.03 | 1 | 0.861 |
| PSQI sleep disturbance | PSQI sleep quality | 8.83 | 1 | **0.003** |
| PSQI sleep disturbance | PSQI sleep medication | 1.25 | 1 | 0.264 |
| PSQI sleep latency | PSQI daytime dysfunction | 8.07 | 1 | **0.005** |
| PSQI sleep latency | PSQI sleep efficiency | 4.55 | 1 | **0.033** |
| PSQI sleep latency | PSQI sleep quality | 2.01 | 1 | 0.156 |
| PSQI sleep latency | PSQI sleep medication | 2.86 | 1 | 0.091 |
| PSQI daytime dysfunction | PSQI sleep efficiency | 0.76 | 1 | 0.383 |
| PSQI daytime dysfunction | PSQI sleep quality | 3.71 | 1 | 0.054 |
| PSQI daytime dysfunction | PSQI sleep medication | 2.77 | 1 | 0.096 |
| PSQI sleep efficiency | PSQI sleep quality | 6.00 | 1 | **0.014** |
| PSQI sleep efficiency | PSQI sleep medication | 3.63 | 1 | 0.057 |
| PSQI sleep efficiency | PSQI sleep medication | 1.51 | 1 | 0.219 |

*df*, degrees of freedom; *PSQI*, Pittsburgh Sleep Quality Index

| **Supplemental Table 2. Independent t-tests for variables with significant correlations (*N* = 173)** | | | | |
| --- | --- | --- | --- | --- |
| Variable 1 | Variable 2 | *t* | *df* | p-value |
| Educational attainment | MoCA score | -2.217 | 171 | 0.028 |
| Educational attainment | Verbal paired associates memory | -3.475 | 171 | 0.001 |
| Educational attainment | Overall memory score | -2.185 | 171 | 0.030 |

*df*, degrees of freedom; *MoCA*, Montreal Cognitive Assessment

| **Supplementary Table 3. Linear regression of associations between sleep and visual paired associates memory using continuous PSQI scores (*N* = 173)** | | | | |
| --- | --- | --- | --- | --- |
|  | Model 1a | Model 1b | Model 2a | Model 2b |
|  | *ϐ* (SE) | *ϐ* (SE) | *ϐ* (SE) | *ϐ* (SE) |
| PSQI global score | -.009 | -.008 |  |  |
|  | (.025) | (.025) |  |  |
| Age (mean-centered) | -.162* | -.394* | -.133 | -.363 |
|  | (.009) | (.018) | (.009) | (.023) |
| PSQI global score × Age |  | .263 |  |  |
|  |  | (.003) |  |  |
| Sex | .035 | .022 | .033 | .020 |
| (ref. male) | (.146) | (.146) | (.148) | (.149) |
| Educational attainment | -.026 | -.029 | -.078 | -.092 |
| (ref. up to some college) | (.168) | (.167) | (.173) | (.174) |
| MoCA score | .307*** | .313*** | .324*** | .300*** |
|  | (.033) | (.032) | (.033) | (.034) |
| PSQI sleep duration |  |  | -.155 | -.158 |
|  |  |  | (.134) | (.133) |
| PSQI sleep disturbance |  |  | -.005 | .003 |
|  |  |  | (.143) | (.152) |
| PSQI sleep latency |  |  | -.087 | -.116 |
|  |  |  | (.088) | (.091) |
| PSQI daytime dysfunction |  |  | -.014 | .006 |
|  |  |  | (.109) | (.110) |
| PSQI sleep efficiency |  |  | -.044 | .004 |
|  |  |  | (.098) | (.100) |
| PSQI sleep quality |  |  | .178 | .155 |
|  |  |  | (.127) | (.129) |
| PSQI sleep medication |  |  | .069 | .040 |
|  |  |  | (.073) | (.075) |
| PSQI sleep duration × Age |  |  |  | .176 |
|  |  |  |  | (.016) |
| PSQI sleep disturbance × Age |  |  |  | .076 |
|  |  |  |  | (.017) |
| PSQI sleep latency × Age |  |  |  | .018 |
|  |  |  |  | (.012) |
| PSQI daytime dysfunction × Age |  |  |  | .101 |
|  |  |  |  | (.014) |
| PSQI sleep efficiency × Age |  |  |  | -.195* |
|  |  |  |  | (.013) |
| PSQI sleep quality × Age |  |  |  | -.040 |
|  |  |  |  | (.016) |
| PSQI sleep medication × Age |  |  |  | .176* |
|  |  |  |  | (.010) |

**p* < .05, *** *p* < .001

*ϐ*, standardized beta coefficient; *SE*, standard errors; *ref*., reference group; *MoCA*, Montreal Cognitive Assessment; *PSQI*, Pittsburgh Sleep Quality Index

| **Supplementary Table 4. Linear regression of associations between sleep and verbal paired associates memory using continuous PSQI scores (*N* = 173)** | | | | |
| --- | --- | --- | --- | --- |
|  | Model 1a | Model 1b | Model 2a | Model 2b |
|  | *ϐ* (SE) | *ϐ* (SE) | *ϐ* (SE) | *ϐ* (SE) |
| PSQI global score | .143* | .142* |  |  |
|  | (.024) | (.024) |  |  |
| Age (mean-centered) | -.131 | -.022 | -.113 | -.017 |
|  | (.009) | (.018) | (.009) | (.022) |
| PSQI global score × Age |  | -.123 |  |  |
|  |  | (.003) |  |  |
| Sex | .073 | .079 | .056 | .062 |
| (ref. male) | (.141) | (.142) | (.143) | (.147) |
| Educational attainment | .230** | .232** | .207** | .204** |
| (ref. up to some college) | (.162) | (.162) | (.167) | (.171) |
| MoCA score | .343*** | .340*** | .363*** | .344*** |
|  | (.031) | (.032) | (.032) | (.033) |
| PSQI sleep duration |  |  | .007 | .009 |
|  |  |  | (.129) | (.131) |
| PSQI sleep disturbance |  |  | -.052 | -.057 |
|  |  |  | (.137) | (.150) |
| PSQI sleep latency |  |  | .056 | .052 |
|  |  |  | (.085) | (.090) |
| PSQI daytime dysfunction |  |  | .111 | .100 |
|  |  |  | (.105) | (.108) |
| PSQI sleep efficiency |  |  | -.126 | -.132 |
|  |  |  | (.094) | (.099) |
| PSQI sleep quality |  |  | .143 | .156 |
|  |  |  | (.123) | (.127) |
| PSQI sleep medication |  |  | .126 | .145* |
|  |  |  | (.071) | (.074) |
| PSQI sleep duration × Age |  |  |  | .103 |
|  |  |  |  | (.016) |
| PSQI sleep disturbance × Age |  |  |  | .012 |
|  |  |  |  | (.017) |
| PSQI sleep latency × Age |  |  |  | -.091 |
|  |  |  |  | (.011) |
| PSQI daytime dysfunction × Age |  |  |  | .055 |
|  |  |  |  | (.014) |
| PSQI sleep efficiency × Age |  |  |  | -.025 |
|  |  |  |  | (.013) |
| PSQI sleep quality × Age |  |  |  | -.181 |
|  |  |  |  | (.015) |
| PSQI sleep medication × Age |  |  |  | -.007 |
|  |  |  |  | (.010) |

**p* < .05, ** *p* < .01, *** *p* < .001

*ϐ*, standardized beta coefficient; *SE*, standard errors; *ref*., reference group; *MoCA*, Montreal Cognitive Assessment; *PSQI*, Pittsburgh Sleep Quality Index

| **Supplementary Table 5. Linear regression of associations between sleep and MST LDI using continuous PSQI scores (*N* = 173)** | | | | |
| --- | --- | --- | --- | --- |
|  | Model 1a | Model 1b | Model 2a | Model 2b |
|  | *ϐ* (SE) | *ϐ* (SE) | *ϐ* (SE) | *ϐ* (SE) |
| PSQI global score | -.111 | -.111 |  |  |
|  | (.024) | (.024) |  |  |
| Age (mean-centered) | -.257*** | -.205 | -.262*** | -.211 |
|  | (.009) | (.018) | (.009) | (.022) |
| PSQI global score × Age |  | -.058 |  |  |
|  |  | (.003) |  |  |
| Sex | .042 | .045 | .054 | .055 |
| (ref. male) | (.140) | (.141) | (.141) | (.143) |
| Educational attainment | .006 | .007 | -.020 | -.029 |
| (ref. up to some college) | (.161) | (.161) | (.164) | (.168) |
| MoCA score | .352*** | .351*** | .358*** | .340*** |
|  | (.031) | (.031) | (.031) | (.032) |
| PSQI sleep duration |  |  | .002 | -.011 |
|  |  |  | (.128) | (.129) |
| PSQI sleep disturbance |  |  | .091 | .100 |
|  |  |  | (.136) | (.146) |
| PSQI sleep latency |  |  | -.079 | -.113 |
|  |  |  | (.084) | (.088) |
| PSQI daytime dysfunction |  |  | -.096 | -.078 |
|  |  |  | (.103) | (.106) |
| PSQI sleep efficiency |  |  | -.101 | -.054 |
|  |  |  | (.093) | (.097) |
| PSQI sleep quality |  |  | -.095 | -.105 |
|  |  |  | (.121) | (.125) |
| PSQI sleep medication |  |  | .124 | .094 |
|  |  |  | (.070) | (.072) |
| PSQI sleep duration × Age |  |  |  | .072 |
|  |  |  |  | (.013) |
| PSQI sleep disturbance × Age |  |  |  | -.011 |
|  |  |  |  | (.016) |
| PSQI sleep latency × Age |  |  |  | .024 |
|  |  |  |  | (.011) |
| PSQI daytime dysfunction × Age |  |  |  | -.009 |
|  |  |  |  | (.014) |
| PSQI sleep efficiency × Age |  |  |  | -.207* |
|  |  |  |  | (.013) |
| PSQI sleep quality × Age |  |  |  | -.053 |
|  |  |  |  | (.015) |
| PSQI sleep medication × Age |  |  |  | .104 |
|  |  |  |  | (.010) |

**p* < .05, *** *p* < .001

*ϐ*, standardized beta coefficient; *SE*, standard errors; *ref*., reference group; *MoCA*, Montreal Cognitive Assessment; *PSQI*, Pittsburgh Sleep Quality Index; *MST,* Mnemonic Similarity Task; *LDI,* Lure Discrimination Index

| **Supplementary Table 6. Linear regression of associations between sleep and overall memory score using continuous PSQI scores (*N* = 173)** | | | | |
| --- | --- | --- | --- | --- |
|  | Model 1a | Model 1b | Model 2a | Model 2b |
|  | *ϐ* (SE) | *ϐ* (SE) | *ϐ* (SE) | *ϐ* (SE) |
| PSQI global score | .010 | .010 |  |  |
|  | (.017) | (.017) |  |  |
| Age (mean-centered) | -.238*** | -.267 | -.221*** | -.254 |
|  | (.006) | (.013) | (.006) | (.016) |
| PSQI global score × Age |  | .033 |  |  |
|  |  | (.002) |  |  |
| Sex | .065 | .063 | .062 | .060 |
| (ref. male) | (.102) | (.103) | (.103) | (.104) |
| Educational attainment | .092 | .092 | .048 | .037 |
| (ref. up to some college) | (.117) | (.118) | (.120) | (.122) |
| MoCA score | .434*** | .435*** | .453*** | .426*** |
|  | (.023) | (.023) | (.023) | (.023) |
| PSQI sleep duration |  |  | -.062 | -.068 |
|  |  |  | (.093) | (.093) |
| PSQI sleep disturbance |  |  | .015 | .020 |
|  |  |  | (.099) | (.106) |
| PSQI sleep latency |  |  | -.047 | -.076 |
|  |  |  | (.061) | (.064) |
| PSQI daytime dysfunction |  |  | .000 | .012 |
|  |  |  | (.075) | (.077) |
| PSQI sleep efficiency |  |  | -.118 | -.079 |
|  |  |  | (.068) | (.070) |
| PSQI sleep quality |  |  | .096 | .088 |
|  |  |  | (.088) | (.090) |
| PSQI sleep medication |  |  | .139* | .122 |
|  |  |  | (.051) | (.053) |
| PSQI sleep duration × Age |  |  |  | .151 |
|  |  |  |  | (.011) |
| PSQI sleep disturbance × Age |  |  |  | .033 |
|  |  |  |  | (.012) |
| PSQI sleep latency × Age |  |  |  | -.022 |
|  |  |  |  | (.008) |
| PSQI daytime dysfunction × Age |  |  |  | .063 |
|  |  |  |  | (.010) |
| PSQI sleep efficiency × Age |  |  |  | -.184* |
|  |  |  |  | (.009) |
| PSQI sleep quality × Age |  |  |  | -.119 |
|  |  |  |  | (.011) |
| PSQI sleep medication × Age |  |  |  | .117 |
|  |  |  |  | (.007) |

**p* < .05, *** *p* < .001

*ϐ*, standardized beta coefficient; *SE*, standard errors; *ref*., reference group; *MoCA*, Montreal Cognitive Assessment; *PSQI*, Pittsburgh Sleep Quality Index
